# Sports Cardiology: From Diagnosis to Clinical Management—Towards Individualized Cardiovascular Care Through Exercise

**DOI:** 10.3390/jcdd13090434

**Published:** 2026-09-03

**Authors:** Christos Kourek, Eleftherios Karatzanos, Stavros Dimopoulos

**Affiliations:** 1 Clinical Ergospirometry, Exercise and Rehabilitation Laboratory, National and Kapodistrian University of Athens, 106 75 Athens, Greece; chris.kourek.92@gmail.com (C.K.); lkaratzanos@gmail.com (E.K.); 2 Department of Cardiac Surgery Research, Lankenau Institute for Medical Research, Wynnewood, PA 19096, USA; 3 Cardiac Surgery ICU, Onassis Cardiac Surgery Center, 176 74 Athens, Greece

This Special Issue, “Sports Cardiology: From Diagnosis to Clinical Management”, was developed to reflect the expanding scope of sports cardiology, a field that now extends well beyond the assessment of competitive athletes alone. The articles included in this collection address cardiovascular evaluation across a broad clinical spectrum, ranging from preparticipation screening and physiological adaptation to exercise to congenital heart disease, heart failure, diabetes, and exercise-based rehabilitation in critically ill patients [1,2,3,4,5,6,7,8]. The purpose of this closing Editorial is to bring these contributions into a common clinical perspective and to emphasize how exercise may serve, depending on the setting, as a diagnostic challenge, a tool for risk stratification, a marker of functional reserve, and a therapeutic intervention. Contemporary sports cardiology increasingly requires this broader and more individualized approach, combining clinical assessment, exercise testing, cardiovascular imaging, disease-specific risk evaluation, and shared decision-making in order to promote safe participation in physical activity without unnecessary restriction [9,10,11].

An appropriate starting point is preparticipation cardiovascular screening. Jiravska Godula et al. evaluated family history within four widely used screening systems in 13,876 athletes and found that 1.28% had a positive family history of sudden cardiac death (SCD) or cardiovascular disease according to at least one protocol [7]. Importantly, the proportion classified as positive varied according to the questionnaire used, being the highest with PPE-4 and progressively lower with FIFA, AHA, and IOC criteria. The study therefore highlights a simple but frequently underestimated problem: even a basic component of cardiovascular screening such as family history is not necessarily captured uniformly. A family history should not be treated as a one-time administrative question, but as information that may evolve as relatives age or new diagnoses emerge. This is particularly relevant because preparticipation evaluation aims to identify the small subgroup of athletes in whom additional investigation may prevent potentially serious events, while avoiding unnecessary testing or restriction in the much larger low-risk population [12,13,14,15,16,17,18]. The association observed between a positive family history and a higher maximal exercise heart rate is interesting, but its clinical significance remains uncertain and requires longitudinal evaluation. Equally relevant was the relatively low representation of female athletes, emphasizing the need for screening strategies that are adequately validated across both sexes [7].

Once major cardiovascular disease has been excluded, another challenge emerges: distinguishing normal or even advantageous exercise-related adaptation from findings that may represent cardiovascular risk. Pellegrino et al. examined this question from the vascular perspective in 240 male athletes [3]. Soccer players differed from runners in ankle–brachial index (ABI), arterial stiffness, blood pressure, and maximal aerobic capacity, while mediation analyses suggested that ABI and the cardio-ankle vascular index (CAVI) contributed to differences in exercise performance. These observations extend the evaluation of the athlete beyond conventional resting blood pressure and cardiac imaging and suggest that peripheral vascular characteristics may reflect, at least partly, the type and intensity of habitual training [3]. Arterial stiffness is increasingly recognized as an integrative marker of vascular function, but exercise modality itself can influence arterial properties, with endurance and resistance training producing different vascular adaptations [19,20,21,22]. For this reason, values obtained in highly trained individuals should always be interpreted within the context of sport discipline, training exposure, age, and cardiovascular phenotype rather than against population reference values alone.

The study by Rovithis et al. addresses a related question at the myocardial level [4]. Using isometric handgrip stress echocardiography, the investigators compared 10 physically active men with 10 sedentary middle-aged men. Although resting diastolic indices were broadly similar, the response to acute afterload differed: active participants maintained a more favorable E/e′ response, whereas sedentary individuals demonstrated an increase in E/e′ during peak handgrip exercise. The findings suggest that regular physical activity may be associated with more favorable left ventricular lusitropic adaptation during an acute hemodynamic challenge [4]. The study is small and limited to men, and the isolated interpretation of E/e′ has recognized limitations; nevertheless, it illustrates why dynamic testing may add information that resting measurements cannot provide. Exercise-related cardiac adaptation should not be considered only in terms of chamber size or wall thickness. Myocardial relaxation, ventricular compliance, filling pressures, and the ability of the heart to accommodate an abrupt increase in loading conditions also form part of the physiological phenotype of the trained heart [23,24,25,26,27]. Stress echocardiography may therefore have a broader role in sports cardiology when resting findings are inconclusive or symptoms occur primarily during exertion.

This concept of assessing the cardiovascular system dynamically rather than exclusively at rest is further developed by Zimmermann et al. [8]. In an exploratory analysis of eight asymptomatic adults with type 1 diabetes mellitus (T1D), two-dimensional speckle-tracking echocardiography was performed before and after peak bicycle exercise. Left ventricular contractile reserve was preserved, and no significant adverse dynamic change in left or right ventricular strain was identified. In contrast, the phasic left atrial contractile deformation pattern changed significantly after exercise [8]. Given the very small sample and absence of a matched healthy control group, these findings should remain hypothesis-generating rather than being interpreted as evidence of an early diabetic cardiomyopathy phenotype. Nevertheless, they demonstrate the feasibility of combining exercise with deformation imaging to investigate subclinical myocardial abnormalities. Speckle-tracking techniques have already shown that conventional ejection fraction may fail to identify early myocardial dysfunction in diabetes, while left atrial mechanics may provide complementary information regarding ventricular filling and atrioventricular coupling [28,29,30,31,32,33,34]. Whether post-exercise deformation imaging can improve early risk stratification beyond resting echocardiography remains an important question for future prospective studies.

Congenital heart disease provides perhaps one of the clearest examples of how sports cardiology has shifted from restriction toward individualized assessment. Yang et al. examined serial cardiopulmonary exercise testing (CPET) and echocardiographic findings in patients with pulmonary stenosis (PS) who had undergone appropriate intervention or clinical follow-up [6]. Exercise capacity in properly managed patients was comparable to that of matched healthy individuals, and no significant deterioration in absolute peak oxygen consumption was demonstrated during follow-up. At the same time, declining trends in percentage-predicted aerobic capacity and pulmonary function were observed during adolescence, supporting continued surveillance rather than assuming that successful anatomical treatment permanently resolves all functional concerns [6]. This distinction is clinically important. Patients with congenital abnormalities may feel well and have reassuring resting hemodynamics while subtle functional limitations become evident only during exercise. Serial CPET can therefore help clinicians move from lesion-based recommendations to objective, patient-specific exercise advice [35,36,37,38,39,40].

Kauling et al. extend this principle into an especially demanding environment: recreational SCUBA diving in adults with congenital heart disease (ACHD) [5]. Historically, many individuals with ACHD have been advised against diving, frequently on the basis of limited direct evidence. The authors provide a structured fitness-to-dive approach incorporating clinical assessment, electrocardiography, echocardiography, exercise testing, and the specific residual anatomy and physiology of the congenital lesion. Conditions associated with right-to-left shunting, cyanosis, significant ventricular dysfunction, severe obstruction, important valvular disease, relevant aortic dilatation, or unstable arrhythmias may remain incompatible with diving, whereas selected patients with successfully repaired lesions may be considered after detailed evaluation [5]. This is consistent with the wider direction of contemporary ACHD care, in which exercise recommendations are increasingly based on ventricular function, pulmonary pressure, aortic dimensions, arrhythmia burden, oxygen saturation, and functional capacity rather than diagnosis alone [35,36,37,38,39,40,41,42]. Diving also reminds us that “exercise” is not a uniform exposure: environmental pressure, cold, immersion, equipment load, and the consequences of sudden incapacitation can fundamentally modify cardiovascular risk. Shared decision-making must therefore include the characteristics of the sport itself, not only those of the patient.

This Special Issue also emphasizes that sports cardiology and exercise medicine extend far beyond apparently healthy competitive athletes. In patients with heart failure (HF), exercise is an established therapeutic intervention and may influence cardiovascular biology at the cellular level. Kourek et al. reviewed the effects of acute and chronic exercise on circulating endothelial cells and endothelial progenitor cells (EPCs) in HF [2]. The available studies generally support an exercise-mediated increase in the number and functional capacity of EPC populations, potentially through complementary shear-stress and hypoxic/ischemic signaling pathways. This may contribute to endothelial repair, angiogenesis, microvascular function, and ultimately the favorable peripheral adaptations associated with cardiac rehabilitation [2]. Exercise training is firmly incorporated into contemporary HF management, with improvements in functional capacity and quality of life demonstrated across multiple studies [43,44,45,46,47]. The EPC literature adds a mechanistic dimension to these clinical benefits, although substantial methodological heterogeneity remains in cellular definition and quantification. Standardized phenotyping and multicenter studies are needed before EPC responses can become a practical biomarker for individualized exercise prescription [48,49].

The review of exercise training during extracorporeal membrane oxygenation (ECMO) by Kourek et al. takes the therapeutic role of exercise to an even more critically ill population [1]. At first glance, rehabilitation during ECMO may appear remote from conventional sports cardiology. In reality, it represents the same fundamental clinical concept at the opposite end of the functional spectrum: physical activity should be prescribed according to physiological capacity, risk, and therapeutic objective rather than withheld solely because cardiovascular or cardiorespiratory disease is severe. Early mobilization, cycling, resistance exercise, neuromuscular electrical stimulation, respiratory training, and progressive ambulation have all been used in carefully selected ECMO patients, particularly as a bridge to transplantation or recovery [1,50,51,52,53,54,55,56]. However, this is not routine exercise training. Cannulation, bleeding risk, hemodynamic instability, arrhythmias, hypoxemia, vasopressor requirements, and device-related complications require continuous assessment. The review correctly emphasizes that rehabilitation should occur in experienced centers under the supervision of a multidisciplinary ECMO team and that individualized protocols require further validation [1]. The broader message is important: even in advanced disease, immobility has consequences, and exercise may remain part of treatment when appropriately adapted.

Taken together, the eight contributions of this Special Issue [1,2,3,4,5,6,7,8] illustrate several principles that are increasingly central to sports cardiology. Exercise is simultaneously an exposure, a diagnostic stressor, a marker of functional capacity, and a therapeutic intervention. Resting cardiovascular evaluation is often insufficient; dynamic information derived from CPET, stress echocardiography, vascular assessment, or deformation imaging can reveal physiological responses that are otherwise hidden. At the same time, a cardiovascular diagnosis should not automatically translate into exclusion from physical activity. The relevant question is whether the individual’s disease substrate, functional status, treatment, type of exercise, environmental exposure, and personal objectives can be combined within an acceptably safe strategy. This approach naturally requires periodic reassessment because the athlete, the disease, and the exercise exposure may all change over time.

Several limitations across the published studies also indicate where the field must move next. Small single-center cohorts, male predominance, retrospective designs, heterogeneous definitions, limited longitudinal outcome data, and scarcity of randomized trials remain common. Future sports cardiology research should therefore prioritize prospective multicenter registries, greater inclusion of female athletes, standardized preparticipation screening instruments, serial CPET-based phenotyping, integration of advanced imaging with clinical outcomes, and exercise protocols tailored to specific diseases rather than broadly defined cardiovascular populations. Importantly, new diagnostic parameters should demonstrate not only statistical differences but also that they improve clinical decisions, safety, exercise prescription, or patient-centered outcomes.

In closing this Special Issue, the published contributions demonstrate how broad sports cardiology has become [1,2,3,4,5,6,7,8]. The field now connects the young athlete undergoing preparticipation evaluation with the adult with congenital heart disease who wishes to dive, the patient with T1D undergoing advanced exercise imaging, the individual with HF entering cardiac rehabilitation, and even the critically ill patient beginning mobilization while receiving extracorporeal support. Despite their obvious differences, these scenarios share the same clinical objective: to identify cardiovascular risk accurately without unnecessarily depriving patients of the benefits of physical activity and to use exercise, whenever possible, as part of the treatment rather than merely as a potential hazard. We hope that the work presented in this Special Issue will encourage further research toward more precise diagnosis, safer participation, and truly individualized exercise-based cardiovascular care.
